# Supplementary material for: Integrated analysis of the aqueous humor microbiome and lens capsule transcriptome in high myopia cataract: a pilot study
Source: Front Med (Lausanne). 2026 Jun 16;13:1845205. doi: 10.3389/fmed.2026.1845205 (PMC13314463; doi:10.3389/fmed.2026.1845205)

euclidean distance

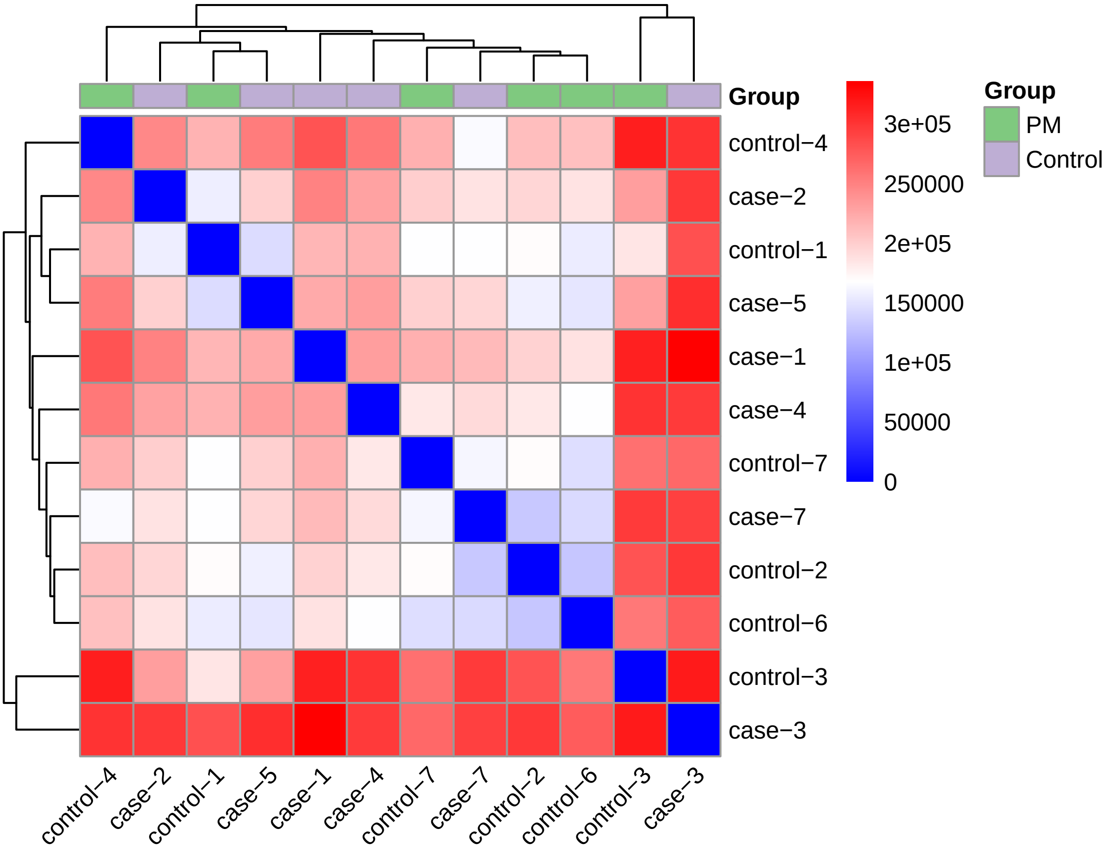

Supplement: Supplementary file 3 [file Data_Sheet_1.zip › 3.Beta_Diversity/Distance/C372089/euclidean_distance.pdf]
